# Manganese-Loaded Liposomes: An In Vitro Study for Possible Diagnostic Application

**DOI:** 10.3390/molecules29143407

**Published:** 2024-07-20

**Authors:** Maddalena Sguizzato, Petra Martini, Francesca Ferrara, Lorenza Marvelli, Markus Drechsler, Giovanni Reale, Francesca Calderoni, Federica Illuminati, Francesca Porto, Giorgia Speltri, Licia Uccelli, Melchiore Giganti, Alessandra Boschi, Rita Cortesi

**Affiliations:** 1 Department of Chemical, Pharmaceutical and Agricultural Sciences (DoCPAS), University of Ferrara, 44121 Ferrara, Italy; maddalena.sguizzato@unife.it (M.S.); francesca.ferrara@unife.it (F.F.); lorenza.marvelli@unife.it (L.M.); giorgia.speltri@unife.it (G.S.); rita.cortesi@unife.it (R.C.); 2 Biotechnology Inter University Consortium (C.I.B.), Ferrara Section, University of Ferrara, 44121 Ferrara, Italy; 3 Department of Environmental and Prevention Sciences, University of Ferrara, 44121 Ferrara, Italy; petra.martini@unife.it; 4 Bavarian Polymer Institute Keylab “Electron and Optical Microscopy”, University of Bayreuth, 95447 Bayreuth, Germany; markus.drechsler@uni-bayreuth.de; 5 Department of Translational Medicine, University of Ferrara, 44121 Ferrara, Italy; giovanni.reale@unife.it (G.R.); francesca.porto@unife.it (F.P.); licia.uccelli@unife.it (L.U.); melchiore.giganti@unife.it (M.G.); 6 Medical Physics Unit, University Hospital of Ferrara, 44124 Ferrara, Italy; francesca.calderoni@ospfe.it; 7 Radiology Unit, University Hospital of Ferrara, 44124 Ferrara, Italy; f.illuminati@ospfe.it

**Keywords:** manganese, liposomes, diagnostic application, MEMRI, contrast agents

## Abstract

The present study investigates the possible use of manganese (Mn)-based liposomal formulations for diagnostic applications in imaging techniques such as magnetic resonance imaging (MRI), with the aim of overcoming the toxicity limitations associated with the use of free Mn^2+^. Specifically, anionic liposomes carrying two model Mn(II)-based compounds, MnCl_2_ (MC) and Mn(HMTA) (MH), were prepared and characterised in terms of morphology, size, loading capacity, and in vitro activity. Homogeneous dispersions characterised mainly by unilamellar vesicles were obtained; furthermore, no differences in size and morphology were detected between unloaded and Mn-loaded vesicles. The encapsulation efficiency of MC and MH was evaluated on extruded liposomes by means of ICP-OES analysis. The obtained results showed that both MC and MH are almost completely retained by the lipid portion of liposomes (LPs), with encapsulation efficiencies of 99.7% for MC and 98.8% for MH. The magnetic imaging properties of the produced liposomal formulations were investigated for application in a potential preclinical scenario by collecting magnetic resonance images of a phantom designed to compare the paramagnetic contrast properties of free MC and MH compounds and the corresponding manganese-containing liposome dispersions. It was found that both LP-MC and LP-MH at low concentrations (0.5 mM) show better contrast (contrast-to-noise ratios of 194 and 209, respectively) than solutions containing free Mn at the same concentrations (117 and 134, respectively) and are safe to use on human cells at the selected dose. Taken together, the results of this comparative analysis suggest that these liposome-containing Mn compounds might be suitable for diagnostic purposes.

## 1. Introduction

The most commonly used contrast agents (CAs) exploit a property of the lanthanide ion gadolinium (Gd) known as paramagnetism. CAs based on the Gd ion are intravenously administered to enhance the signal in MR imaging [1].

Gd is a natural element [2] but is toxic for humans; therefore, the ion Gd^3+^ is bound or chelated to a larger electron-donating molecule or ligand [3,4] to make it suitable for human administration and safe for in vivo use in most circumstances [2]. Although nephrogenic systemic fibrosis (NSF) has almost been completely eradicated with modern MRI techniques, additional concerns about the safety of Gd-based CAs have recently emerged, especially following the discovery that the repeated exposure to these chelates can lead to the deposition of Gd in the basal ganglia [5].

Manganese (Mn) is an essential trace element involved in bone development as an enzyme activator. It also plays roles in neurological, immunological, and endocrine activities and, through superoxide dismutase, helps protect mitochondrial membranes from oxidative stress [6].

Unlike Gd, Mn naturally occurs in metabolic excretion pathways, providing a safer metabolic profile [5]. Furthermore, due to its involvement in mitochondrial activity, Mn is an optimal contrast agent for the MR imaging of organs rich in mitochondria such as the liver, pancreas, heart, and kidneys [7].

Mn-based contrast agents are particularly promising for clinical diagnostic imaging, including multimodal techniques. They offer several advantages, such as the availability of radioactive isotopes suitable for PET imaging. One such isotope is ^52^Mn, which has a half-life of 5.6 days and a β^+^ decay with 29.4% abundance and an average energy (E_aveβ+_) of 0.24 MeV. These properties make Mn ions suitable for the developing MRI/PET dual-mode imaging contrast agents. The combination of PET and MRI modalities enables an improved visualisation and characterisation of tissues, providing complementary information. Overall, the use of Mn-based CAs shows potential for dual-mode imaging applications, enhancing imaging capabilities while potentially addressing safety concerns associated with traditional Gd-based CAs.

The formulations of Mn-based CAs delineate their behaviour in vivo and their possible related toxicity [4]. Manganese, possessing paramagnetic properties, is able to decrease the relaxation time T1 of water protons, thereby increasing Mn-enhanced magnetic resonance imaging (MEMRI) especially in T1-weighted images. The correlation between MRI relaxation times and the concentration of Mn(II) ions in tissues highlights a direct proportionality: higher concentrations of Mn(II) result in stronger and more discernible contrast, thereby enhancing image quality [8]. However, the primary obstacle to the clinical application of Mn(II) ions as a contrast agent remains their neurotoxic effects [8]. Therefore, the key consideration in using Mn as a contrast agent lies in minimising adverse effects by employing the lowest effective dosage detectable by MRI.

Regarding liposomes, they are extensively discussed in the literature as blood-pool CAs [7]. Liposome-based contrast agents encapsulate the signal-generating component within their central core [9] and predominantly remain within the vascular space as intravascular agents. This characteristic makes them potentially preferable for quantitative perfusion imaging [2].

Among CAs approved for MRI studies, Table 1 reports their r1 values measured by Rohrer et al. using a clinical MRI scanner at 1.5 T [10].

Intravascular CAs, as described by [9], have extended circulation half-lives in vivo compared to conventional MRI contrast agents. This property enables excellent imaging in studies requiring prolonged image acquisition times. These agents are particularly valuable for evaluating perfusion in ischemic regions, providing insights into capillary permeability in reperfusion areas, the extent of tumour neovascularization, and the associated permeability alterations [11]. Consequently, blood-pool MRI CAs, as highlighted by [12], offer utility beyond their primary role in angiography. They allow for lower dosing and provide a significantly broader window for image acquisition.

To mitigate potential toxicity concerns, we previously investigated the performance of anionic liposomes for delivering a novel class of Mn complexes [13,14]. Our initial assessment of the relaxometric properties of these liposomal formulations revealed high relaxivity values, indicating partial coordination of the Mn(II) centre by its chelators, thereby implicating its involvement in binding to the surface—either inner or outer—of the nanocarrier. It is well established that T1 contrast agents (CAs) must directly interact with the surrounding water protons to affect their relaxation times. In the context of liposomal formulations, this implies that only Mn ions located on the surface are effective.

Furthermore, nanoparticles of smaller size typically exhibit a higher percentage of surface area, resulting in increased r1 relaxivities compared to larger nanoparticles [15].

**Figure 1:**
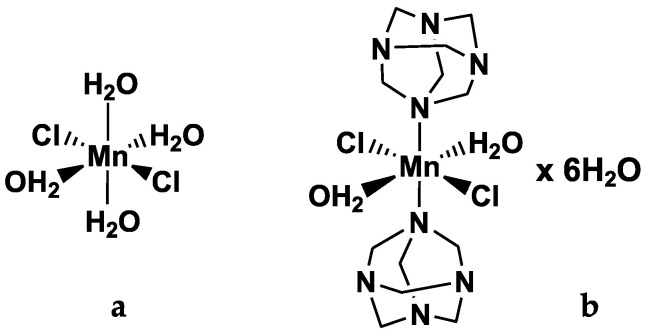
Chemical structure of Mn(II)-based compounds MC (**a**) and MH (**b**). (Original figure).

Following the encouraging results obtained in our previous characterisation study of Mn(II) complexes [13,14,16], this study aims to explore the potential use of manganese ions as a contrast agent while minimising side-effects through encapsulation in liposomes, using the lowest effective MRI-detectable dose. Specifically, this work presents a preliminary clinically oriented evaluation of liposome-encapsulated Mn(II) compounds using a clinical MRI scanner. Significantly, this study evaluates the carrier’s influence on water relaxation times and its impact on endothelial cells, which are crucial in cardiovascular health. A comparison is drawn between two model Mn(II)-based compounds, MnCl_2_ (MC) and Mn(HMTA) (MH) (see Figure 1), in both free-form and liposome-loaded configurations.

## 2. Results and Discussion

### 2.1. Mn Derivatives and Liposome Preparation and Characterization

MH (Figure 1) was prepared and characterised according to the procedure reported in the literature [14,16]. Negatively charged liposomes (LPs) were produced by the hydration method and extrusion [14]. Macroscopically, the liposomal dispersions appear milk-coloured before extrusion and more translucent after extrusion [13,14].

**Figure 2:**
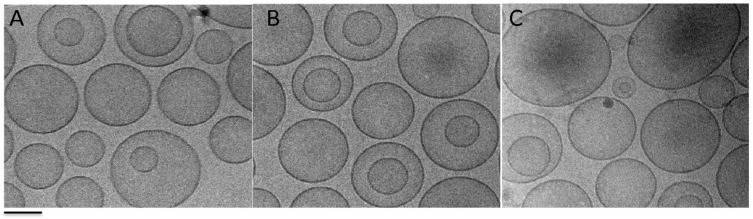
Cryo-TEM images of Mn(II)-loaded liposomes visualised after extrusion. Plain-LP (**A**), LP-MC (**B**), and LP-MH (**C**). Bar corresponds to 100 nm. (Original figure).

The morphology of liposomes after extrusion was investigated by cryo-TEM and the obtained images are shown in Figure 2.

From the analyses of the images, it emerges, as expected, that the extrusion process allowed the obtaining of homogeneous dispersions mainly characterised by the presence of mono- and bi-lamellar liposomes. Furthermore, no differences in size and morphology were evident between unloaded and Mn-loaded vesicles.

The size and charge of liposome dispersions were evaluated in terms of average diameter, polydispersity index, and ζ potential. The values obtained from measurements, performed before and after extrusion through polycarbonate filters with pore sizes of 200 nm, are summarised in Table 2. As expected, after extrusion, the average diameter of liposomes decreases, reflecting the pore size of the filter used for the extrusion. Moreover, the measure of ζ potential confirmed the presence of a net negative surface charge that, in agreement with the literature [17], is slightly affected after the extrusion process. Furthermore, all the formulations showed an absolute ζ potential value greater than 30 mV, ensuring good electrostatic stability of the dispersion [18].

Furthermore, the polydispersity indexes of the extruded preparations were lower than those of the same dispersion before extrusion, suggesting a monomodal distribution and corroborating the morphological results obtained by the cryo-TEM visualisation [19].

The dimensional stability of the produced liposomes was monitored for one month and the obtained data, reported in Table 3, showed that liposomes are stable over time since both the average size and the PdI values remained unchanged during the thirty days of analysis.

The encapsulation efficiency (EE) of MC and MH was evaluated on extruded liposomes by means of ICP-OES analysis. Optical emission spectrometry is an analytical technique used for the quantitative and qualitative determination of metal ions in solution. Particularly, we employed inductively coupled plasma–optical emission spectroscopy (ICP-OES) using the inductively coupled plasma to produce excited atoms and ions emitting electromagnetic radiation at wavelengths characteristic of the element [20]. Loaded liposomes were ultracentrifuged to separate the lipid from the aqueous portions of the preparation [21]. The obtained results showed that both MC and MH are almost completely retained by the lipid portion of LP, indicating an almost quantitative manganese encapsulation in the whole formulation. Specifically, the liposome EE of MC was 99.7%, confirming previous results [13], while in the case of MH, an EE of 98.8%, calculated by applying Equation (1), was found. Based on these results aiming to separate encapsulated from un-encapsulated Mn by the ultracentrifugation method, the complete absence of free Mn in the aqueous phase is evident.

In order to investigate the physical stability of LP in serum, both their size and polydispersity were investigated in the presence of FBS and the dimensional distribution analysis results are summarised in Table 4.

As reported, all formulations maintain their physical stability, in terms of dimensional distribution, when dispersed and stored in FBS. Moreover, comparing the results with those displayed in Table 3, where the stability in water is reported, it is evident that the size and polydispersity values could be considered superimposable. Hence, the liposome stability is not affected by serum composition.

### 2.2. Magnetic Imaging Studies by Means of Phantom

**Figure 3:**
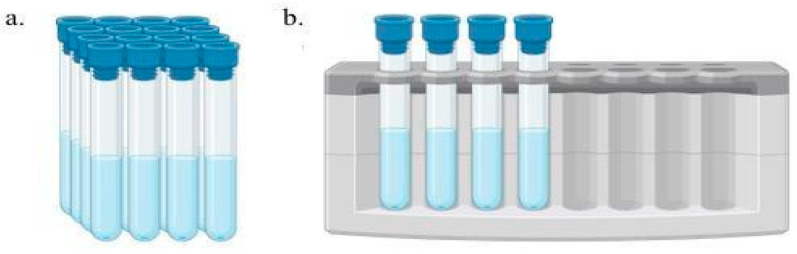
Schematic representation of tube disposition in phantom (**a**) and immersed in water (**b**) (original figure created with BioRender.com, accessed on 13 January 2024).

The aim of this pilot analysis was to gain a preliminary insight into the magnetic imaging properties of these liposomal formulations in a potential preclinical scenario. This study was conducted by collecting magnetic resonance imagery of a phantom, especially designed to compare the paramagnetic contrast properties of MC and MH compounds and the corresponding manganese-containing liposome dispersions (LP-MC and LP-MH). In-serum phantoms were also used to obtain more biologically relevant data. To this aim, the phantom schematically designed in Figure 3 was especially realised to compare the paramagnetic contrast properties of manganese in different media (i.e., water or FBS) either in the free form, as a complex, or loaded in liposomes.

The longitudinal (T1) relaxation times of both free and liposome-loaded formulations in water and in FBS were estimated using a T1 mapping sequence in a 1.5 T MR clinical machine at 20 °C and 37 °C. Relaxation times were measured at different concentrations, and the relaxivities (r1) were obtained from the plot of relaxation rates (R1 = 1/T1) versus complex concentration.

**Figure 4:**
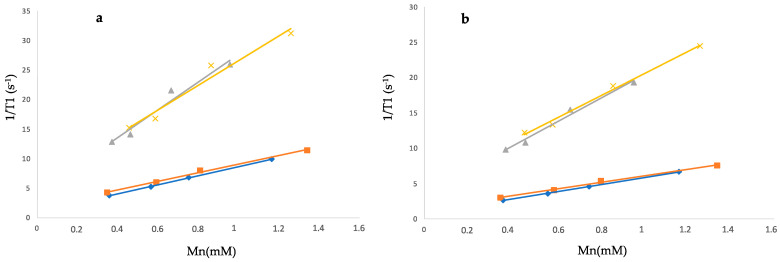
Plot of relaxation rates (R1 = 1/T1) versus complex concentration collected in H_2_O at 20 °C (**a**) and 37 °C (**b**). LP-MC: triangle, LP-MH: cross, MC: diamond, MH: square. For simplicity, the standard deviation values have not been reported. (Original figure).

In Figure 4, the plot of relaxation rates versus Mn concentrations of MC, MH, LP-MC, and LP-MH diluted in H_2_O is reported, whilst in Table 5, the r1 values of Mn formulations either in water or in FBS are summarised. Notably, MC and MH at 1.5 T and at 37 °C showed r1 values of 5.0 and 4.6 mM^−1^s^−1^, respectively, and 7.3 and 7.7 mM^−1^s^−1^ at 20 °C. It should be underlined that these values are superimposable to those obtained in the case of Mn(II) unchelated complexes and are higher than that of the commercially available Gadolinium MRI CA [16].

Notably, the r1 values expressed by Mn-liposome-loaded formulations are threefold higher than those of the free Mn compounds (Table 5). Moreover, all the tested formulations also show significantly higher r1 values than those measured by Rohrer et al. [10] (Table 1).

As expected, the relaxivity for all formulations decreases with the increase in the temperature, indicating the occurrence of a fast exchange condition of bound water molecules [22]. Overall, the relaxivity values of liposome formulations were quite high, in line with paramagnetic centres experiencing slow-tumbling motion, thus clearly suggesting the interaction between the metal centre and the polar portion with a liposomal negative charge. As previously reported [13], this can occur both on the outer surface of the liposomal vesicles and inside the vesicles, promoting the exchange with water molecules compared to what takes place with Mn complexes encapsulated in the lipophilic layer of the liposome [23].

On the other hand, when Mn formulations are poured in FBS, their r1 values show similar relaxivity values for both free and liposome-containing Mn compounds, thus confirming the stable interaction between the metal centre and the liposomal bilayer. It is observed that the relaxivity values of free MC and MH dramatically increase in serum compared to water, probably due to a substantial change in the coordination sphere of the metal core by the chelating groups of serum proteins, whereas the liposomal formulations diluted in serum do not show significant changes in the relaxivity values when they are poured into water or in FBS. This behaviour once again suggests stable Mn encapsulation in liposomes.

The effectiveness of the different Mn-free and liposome-loaded formulations was also evaluated on a sequence routinely used for abdominal contrast-enhanced MRI studies in diagnostic radiology in terms of contrast-to-noise-ratio (CNR).

**Figure 5:**
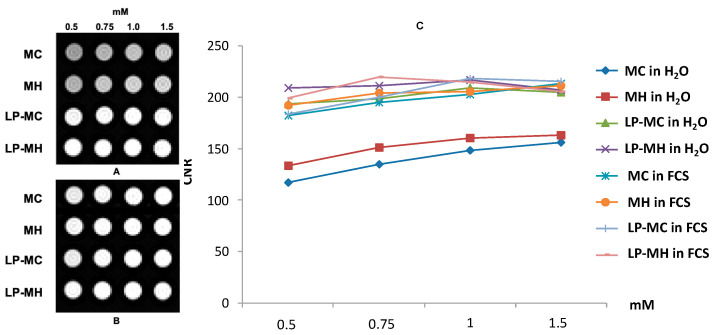
Effectiveness of Mn compounds as free or liposome formulations on a T1-weighted sequence routinely used for abdominal contrast-enhanced MRI studies. VIBE image of the phantom with glass tubes containing different Mn formulations in water (**A**) or in FBS (**B**) at concentrations of 0.5–1.5 mM. For each complex, the contrast-to-noise-ratio (CNR) is plotted as a function of Mn concentrations (**C**). (Original figure).

For T1*w* imaging, a VIBE (Volumetric Interpolated Breath-hold Examination) sequence was used. In addition to qualitative evaluation, the CNR was plotted as a function of concentration for each formulation (Figure 5).

Mn-liposome-loaded formulations show higher CNR values than Mn-free formulations both in water and in FBS. In particular, it is observed that at low concentrations, liposomal formulations already show a better contrast than solutions containing free Mn at the same concentration.

### 2.3. In Vitro Studies

**Figure 6:**
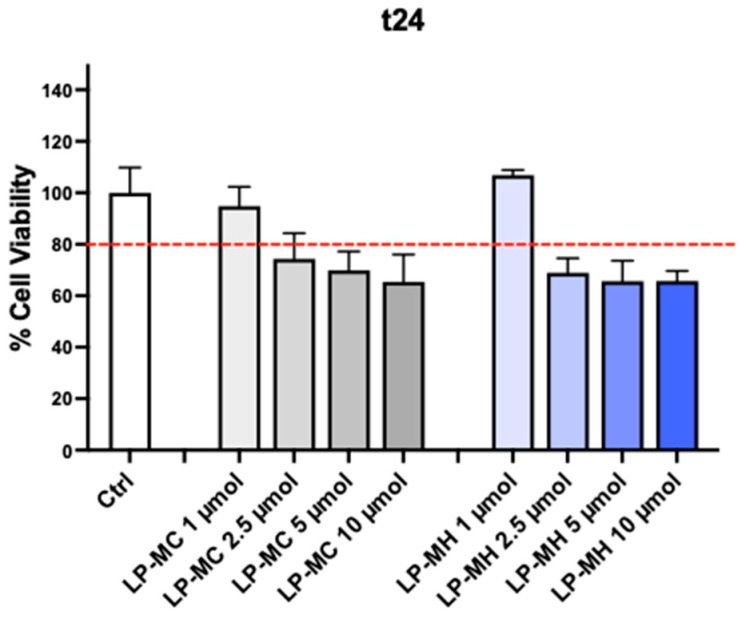
HUVEC cell viability evaluated by MTT test. Cells were treated for 24 h with the indicated doses of LP-MC and LP-MH. The % of cell viability is expressed with respect to untreated control cells (ctrl). The red dash indicates the minimum threshold of cell viability accepted (80% of cell viability). Data are the mean ± SD of three independent experiments with at least three technical replicates each time. (Original figure).

In order to show that these formulations are safe to be used as possible CAs for diagnostic purposes, a cell viability test was assessed on endothelial cells (HUVEC cells) treated with LP-MC or LP-MH at different doses ranging from 1 to 10 μmol. As shown in Figure 6, LP-MC and LP-MH at the lowest dose (i.e., 1 μmol) did not affect cell viability compared to the Ctrl. All the other doses instead led to a decrease in cell viability (below 80%) compared to the Ctrl. Thus, 1 μmol was selected as the dose of Mn to use for the subsequent experiments. Moreover, in order to test the potential toxicity of the Mn-based contrast agents (MnCl_2_ and Mn(HMTA)) on cells after the degradation of liposomes, we assessed an MTT assay by treating HUVEC cells for 24 h with free-Mn(II)-based contrast agents in aqueous solution (i.e., Sol-MC and Sol-MH) at the doses of 1–10 µmol. As shown in Supplementary Figure S1, cell viability was not affected at any concentration. Therefore, it can be supposed that, upon liposome degradation, the released dose of Mn(II)-based contrast agents selected for this study (1 μmol) is safe to use.

**Figure 7:**
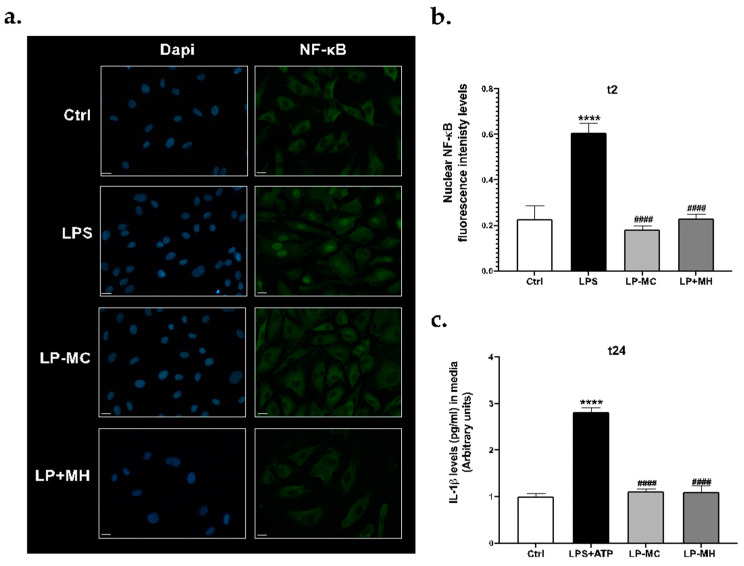
(**a**) Immunofluorescence staining for NF-κB in HUVEC cells treated with LP-MC or LP-MH at the dose of 1 μmol for 2 h. Cells treated with 1 μg/mL of LPS for 2 h were used as the positive control. Green staining represents NF-κB and blue staining (DAPI) represents nuclei. Images were taken at 40× magnification (scale bar = 25 μm). (**b**) The fluorescence levels of NF-κB were quantified with ImageJ software (ImageJ 1.53a) and graphed. (**c**) Released levels of IL-1β in the medium of HUVEC cells treated with LP-MC or LP-MH at the dose of 1 μmol for 24 h. Samples treated with 1 μg/mL of LPS for 24 h + 5 mM of ATP for 2 h were used as the positive control. Data are the results of the averages of at least three different experiments; **** *p* < 0.0001 LPS or LPS+ATP vs. Ctrl; #### *p* < 0.0001 LP-MC or LP-MH vs. LPS or LPS+ATP by 2-way ANOVA followed by Tukey’s post hoc comparison test. (Original figure).

To demonstrate that Mn formulations at the selected dose of 1 μmol did not promote cellular inflammatory damage, HUVEC cells were treated with LP-MC or LP-MH for 2 h and an immunofluorescence staining was assessed to evaluate the activation of the nuclear factor kappa-light-chain-enhancer of activated B cells (NF-κB). Indeed NF-κB is one of the most important modulators of the inflammatory response of the cells and, once activated, it translocates from the cytoplasm to the nucleus to favour the transcription of genes involved in the inflammatory response [24,25]. Precisely, LPS was used as a positive control to show the activation and consequent translocation of NF-κB within the nuclei of cells, whilst as a negative control, untreated HUVEC cells (Ctrl) were considered. The obtained results show that, while the cells treated with LPS, one of the most known inducers of NF-κB, displayed increased levels of nuclear NF-κB, indicating its activation, both LP-MC and LP-MH did not induce NF-κB translocation (Figure 7a). In support of these data, HUVEC cells treated for 24 h with the formulations did not show any release of IL-1β (Figure 7b), one of the main interleukins released during an inflammatory event.

Indeed, the activation of NF-κB has been shown to modulate the inflammatory pathway NLRP3 inflammasome, a multiprotein platform involved in the secretion of inflammatory interleukins, such as IL-1β [26]. Taken together, these results demonstrate that both LP-MC and LP-MH are safe to use on human cells at the selected dose and thus might be suitable for a diagnostic purpose.

## 3. Materials and Methods

### 3.1. Materials

Mn(II)Cl_2_•4H_2_O and hexamethylenetetramine (HMTA) were obtained from Aldrich Chemical (St. Louis, MO, USA). Soybean phosphatidylcholine, namely Phospholipon 90G (PC), was from Lipoid AG (Steinhausen, Switzerland). Cholesterol (CH) and sodium lauroyl lactylate (SLL) were from Merck-Aldrich (Rome, Italy). HUVEC cells (Invitrogen, Carlsbad, CA, USA) were kindly provided by Prof. Voltan of Ferrara University. Solvents of analytical grade were from Merck Serono S.p.A. (Rome, Italy). All other materials and solvents of the high-purity grade were from Sigma-Aldrich (St. Louis, MO, USA). The Mn complex MH was prepared according to the procedure reported in the literature [14,27].

### 3.2. Liposome Preparation and Characterization

Anionic liposomes were prepared as previously described [13] using the hydration method followed by extrusion. A solution of PC, CH, and SLL (4:2:1 mol/mol/mol) in a mixture of CH_2_Cl_2_:CH_3_OH (1:1 *v*/*v*) was evaporated under vacuum (70 bar, rotation speed 3 for 40–45 min) by means of a Rotavapor R-200 (Buchi Italia, Cornaredo, Italy) until obtaining a film on the glass of the flask which was hydrated with an aqueous solution of manganese-based compound (500 μM), swirled by means of a Zx3 vortex mixer (Velp Scientific srl, Usmate Velate, MB, Italy), and subjected to 5 min of sonication in an M2800 ultrasonic bath (Branson CPX-952-238R, VWR International, Milano, Italy). The obtained manganese-loaded liposome, namely LP-MC and LP-MH, underwent fivefold extrusion through two stacked polycarbonate filters with a 0.2 µm pore size (Nucleopore Corp, Pleasanton, CA, USA) supported by a polyester drain disc using an Extruder (Lipex Biomembranes, Vancouver, BC, Canada) and 10–20 bars of nitrogen pressure [13]. After the process, the liposome suspension was collected and stored for further analyses.

Liposome morphology, visualised by means of Cryo-Transmission Electron Microscopy (Cryo-TEM), was evaluated on an aliquot of each liposome dispersion vitrified and transferred to a Zeiss EM922Omega TEM (Zeiss Microscopy GmbH, Jena, Germany) using a cryo-holder (CT3500, Gatan Inc., Pleasanton, CA, USA) [13,28], maintaining the sample temperature under −175 °C under nitrogen flux during the examination and employing doses of approximately 1000–2000 e/nm at 200 kV. Images were recorded digitally and processed using a CCD camera (UltraScan 1000, Gatan Inc., Pleasanton, CA, USA) and GMS 1.4 software (Gatan Inc., Pleasanton, CA, USA), respectively.

Liposome size was measured at 25 °C at an angle of 90° (run time around 180 s) using a Zetasizer Nano S90 (Malvern Instr., Malvern, UK) equipped with a 5 mW helium–neon laser with a wavelength output of 633 nm on aqueous diluted liposome samples (1:20 by volume). The “CONTIN” method [29] was employed to interpret data.

The surface charge of the liposomes was evaluated by measuring the zeta potential (ζ) by means of a Zetasizer Ultra (Malvern Panalytical Ltd., Malvern, UK) instrument. The analyses were conducted at 25 °C in disposable capillary cells (DTS 1080, Malvern) on samples diluted with deionized water (1:20 *v*/*v*). Values were obtained from three independent experiments performed in triplicate.

### 3.3. Evaluation of Manganese Encapsulation by ICP-OES

The encapsulation efficiency (EE) of manganese in liposomes was determined after ultracentrifugation; specifically, 500 μL samples were loaded in a centrifugal filter (Microcon centrifugal filter unit YM-10 membrane, NMWCO 10 kDa, Sigma-Aldrich, St. Louis, MO, USA) and ultracentrifuged at 8000 rpm for 20 min using a SpectrafugeTM 24D Digital Microcentrifuge (Woodbridge, NJ, USA). Then, the lipid phase was analysed with an ICP-OES device (Optima 3100 XL, Perkin-Elmer, Shelton, CT, USA) equipped with an axial torch, segmented array charge-coupled device detector, and Low-Flow GemCone nebulizer with a cyclonic spray chamber for sample introduction, choosing the readings at 259.372 nm among the several wavelengths. Each sample was prepared thrice and subjected to analysis, with the analysed volume being 20 µL. For each condition, the average of the absorbance values obtained was calculated and the manganese concentration was obtained by comparison with a calibration curve obtained after measuring known concentrations of the metal ion [30]. The EE was determined as follows:

EE = [Mn_LIPID_ (mM)/Mn_TOTAL_] × 100 (1)

where Mn_LIPID_ corresponds to the concentration of manganese in the lipid phase measured by ICP-OES and Mn_TOTAL_ is the concentration of manganese used in the formulation.

### 3.4. Magnetic Imaging Studies by Means of Phantom

Different solutions, in terms of manganese content (0.5–1.5 mM), of MC, MH, LP-MC, and LP-MH were obtained by diluting a corresponding 5 mM stock preparation with milliQ H_2_O or a 20% FBS (foetal bovine serum) aqueous solution. Each sample (5 mL), poured into a glass tube (12 × 75 mm), was inserted in a holder and maintained airtight without bubbles. A series of control samples containing empty liposomes in water at the same concentrations in terms of the lipid content of loaded liposomes were also inserted in the phantom (Figure 3). The assembled tubes were immersed in a box filled with water at 20 and 37 °C [31].

MR examinations of the phantom were performed at 1.5 T (MAGNETOM Aera, Siemens Healthcare, Erlangen, Germany) with the use of an abdomen coil. The phantom study aimed to evaluate in a pre-clinical scenario the relaxivity of LP-MC and LP-MH and to compare, both quantitatively and qualitatively, their contrast properties with that of the corresponding manganese compound in the free form. The protocol used included a sequence for T1 mapping through the variable flip angle method and a sequence routinely used for abdominal contrast-enhanced MRI studies.

Default parameters for these sequences were kept:

VIBE (T1 mapping): TR = 4.91 ms, TE = 2.43, ETL = 1, FA = 3–15, voxel = 0.39 mm × 0.39 mm × 3 mm, matrix = 512 × 512;

VIBE: TR = 5.06 ms, TE = 2.43, ETL = 1, FA = 10, voxel = 1.17 mm × 1.17 mm × 3 mm, matrix = 256 × 208.

The images obtained were quantitatively analysed with the software ImageJ (ImageJ 1.44o, U.S. National Institutes of Health, Bethesda, MD, USA) and Matlab (MathWorks, Natick, MA, USA). T1 values were obtained from the map generated by the scanner.

By definition, relaxivities were estimated from Equation (2) through the linear regression of experimental relaxation rates (1/T1) as a function of complex concentration (C).

(1/T1)*obs*(1/T1)*d* + r1[M] (2)

where (1/T1)*obs* and (1/T1)*d* are relaxation rates in the presence and absence of the paramagnetic species, and [M] is the concentration of the paramagnetic species.

To quantitatively evaluate the contrast of the different formulations and concentrations in sequences routinely used for abdominal contrast-enhanced MRI studies, the contrast-to-noise ratio (CNR) was used.

This value was computed as in Equation (3) considering the mean signal intensity (SI) and the standard deviation (SD) within regions of interest placed into the analysed compound and background (BKG).

CNR = (SI_compound_ − SI_BKG_)/SD_BKG_ (3)

### 3.5. In Vitro Studies

#### 3.5.1. Cell Culture and Treatments

Human umbilical vein endothelial cells (HUVEC cells) were cultured in EGM™-2 Endothelial Cell Growth Medium-2 BulletKit™ (cat. CC-3162, Lonza, Basel, Switzerland) in T75 flasks coated with 1 μg/cm^2^ of Fibronectin Bovine Protein Plasma (Cat. 33010018, ThermoFisher Scientific, Waltham, MA, USA) and incubated at 37 °C in 5% CO_2_ and 95% of air. For the experiments, cells were treated with the formulations LP-MC and LP-MH at the indicated doses for 2 or 24 h. An amount of 1 μg/mL of lipopolysaccharide (LPS) or LPS 1 μg/mL+ Adenosine triphosphate (ATP) 5 mM was used as positive controls. Untreated cells (Ctrl) were used as the reference in all experiments. After the different treatments, the samples were collected at the indicated timepoints (t2 or t24) and immunofluorescence staining or ELISA assays were performed.

#### 3.5.2. MTT Assay

HUVEC cells were seeded in a 96-well plate at a density of 5.000 cells/well in 200 μL of EGM™-2 medium. Cells were incubated for 24 h at 37 °C in 5% CO_2_ and 95% air and then treated for 24 h with LP-MC and LP-MH or with the free-Mn(II)-based contrast agents in aqueous solution (i.e., Sol-MC and Sol-MH) at different concentrations of Mn ranging from 1 to 10 μmol. At the end of the 24 h, the treatments were removed and 110 μL of a 0.5 mg/mL MTT solution in EGM™-2 medium was added to each well, according to the manufacture’s protocol (cat. M5655, Sigma-Aldrich, St. Louis, MO, USA). The plate was incubated for 4 h at 37 °C in 5% CO_2_ and the MTT solution was removed carefully to avoid the loss of insoluble purple formazan crystals. The formazan crystals were then dissolved in 100 μL of DMSO at 37 °C for 15 min and the absorbance was measured with a spectrophotometer (Multiskan™ FC Microplate Photometer Cat. 51119000, ThermoFisher Scientific, Waltham, MA, USA) at 590 nm, using 670 nm as the reference wavelength. The absorbance was converted into percentage (%) of viability with respect to untreated cells (Ctrl).

#### 3.5.3. Immunofluorescence Staining

Cells were seeded at a density of 3 × 10^4^ in a 24-well plate onto a 10 mm coverslip previously coated with 1 μg/cm^2^ of Fibronectin Bovine Protein. Cells were incubated overnight at 37 °C in 5% of CO_2_ and then treated for 2 h with LP-MC and LP-MH formulations at the selected dose of 1 μmol. For the positive control, cells were treated with 1 μg/mL of LPS for 2 h to stimulate the activation of nuclear factor kappa-light-chain-enhancer of activated B cells (NF-κB).

After 2 h, coverslips were collected, washed twice in PBS, and fixed with ice-cold methanol for 15 min at 4 °C. Cells were then permeabilized with 0.25% Triton-x in PBS for 15 min at RT, washed 3 times in PBS, and then blocked with 2% of bovine serum albumin (BSA) in PBS for 45 min at RT. Coverslips were incubated overnight at 4 °C with the primary antibody NF-κB D14E12 (Cell Signaling Technology, Danvers, MA, USA) at a dilution of 1:400 in 0.25% BSA in PBS, in a humidified chamber. The day after, cells were incubated with Alexa Fluor Fluorochrome-conjugated secondary antibody (A11008 Alexa Fluor 488 Invitrogen, ThermoFisher Scientific, USA) 1:1000 in PBS-BSA 0.25% for 1 h at RT. After removal of the secondary antibody, DAPI (D1306 Invitrogen ThermoFisher Scientific, Waltham, MA, USA) was used to stain nuclei. Coverslips were mounted onto glass slides using PermaFluorAqueous Mounting Medium (TA-006-FM, ThermoFisher Scientific, Waltham, MA, USA) and examined using a Leica light microscope equipped with epifluorescence at 40× magnification. ImageJ software (ImageJ 1.53a, Wayne Rasband National Institute of Health, Bethesda, MD, USA) was used to quantify the images.

#### 3.5.4. ELISA Assay for IL-1β

The levels of IL-1β were measured in media of HUVEC cells treated with 1 μmol of LP-MC or LP-MH for 24 h. For the positive control, cells were treated with 1 μg/mL of LPS for 24 h and 2 h of ATP 5 mM to stimulate inflammation. IL-1β levels were measured using the IL-1β ELISA kit (Cat. DY201-05, Novus Biologicals, Centennial, CO, USA) according to the manufacturer’s instructions. IL-1β levels are expressed as pg/mL in media. The absorbance was measured at 450 nm using a spectrophotometer (Multiskan™ FC Microplate Photometer Cat. 51119000, ThermoFisher Scientific, Waltham, MA, USA). The reference wavelength was set to 570 nm. IL-1β levels are expressed as pg/mL in media.

#### 3.5.5. Statistical Analysis

For all the experiments, GraphPad Prism 9 software (Version 9.4.1 (458), GraphPad Software Inc., La Jolla, CA, USA) was used to perform the statistical analysis. For each of the variables tested, analysis of variance (1-way ANOVA), followed by Tukey’s post hoc test, was assessed. Data are expressed as the mean ± SD of triplicate determinations obtained in three independent experiments and *p* < 0.05 was considered statistically significant.

## 4. Conclusions

In this study, the possible use of manganese derivatives for diagnostic applications in magnetic resonance imaging was evaluated. Anionic liposomes carrying two model Mn(II)-based compounds, namely MC and MH, were prepared and characterised in terms of morphology, size, loading capacity, and in vitro activity. The magnetic imaging properties of the produced liposomal formulations were investigated for application in a potential preclinical scenario. This study indicates that both LP-MC and LP-MH at low concentrations show better contrast than solutions containing free Mn at the same concentration and are safe to use on human cells at the selected dose, so they might be suitable for diagnostic purposes. Further preclinical and in vivo studies are necessary to investigate stability and the applications of these novel liposomal Mn(II) formulations in clinical medicine. Moreover, towards the aim of achieving an optimal molecular alignment between PET and MRI diagnostic methods, future studies will be dedicated to evaluating the analogous formulations prepared with the ^52^Mn PET radionuclide.

## Figures and Tables

**Table 1:** r1 values (expressed in mM^−1^s^−1^) of CAs approved for MRI in H_2_O or in plasma at 37 °C measured at 1.5 T. The concentration error is ±5% [10].

Name	r1 Values	
	H_2_O	Plasma
Magnevist	3.3	4.1
Gadovist	3.3	5.2
Prohance	2.9	4.1
Multihance	4	6.3
Dotarem	2.9	3.6
Omiscan	3.3	4.3
Teslascan	1.6	3.6
Optimark	3.8	4.7
Resovist	8.7	7.4
Feridex	4.7	4.5
Gadomer	17.3	16
Primovist	4.7	6.9

**Table 2:** Mean size, polydispersity index, and ζ potential of the produced liposomes measured before and after extrusion.

Dispersion Acronym	Before Extrusion			After Extrusion		
	Mean Size (nm ± s.d.)	PdI ± s.d.	ζ Potential (mV ± s.d.)	Mean Size (nm ± s.d.)	PdI ± s.d.	ζ Potential (mV ± s.d.)
**plain-LP**	832.3 ± 12.4	0.35 ± 0.04	−61.12 ± 0.52	176.9 ± 3.3	0.09 ± 0.02	−57.10 ± 0.64
**LP-MC**	698.2 ± 16.3	0.48 ± 0.02	−57.82 ± 1.18	172.9 ± 2.0	0.10 ± 0.01	−67.90 ± 1.04
**LP-MH**	689.1 ± 43.6	0.49 ± 0.04	−59.77 ± 1.04	209.0 ± 3.0	0.11 ± 0.04	−57.15 ± 1.26

**Table 3:** Liposome mean size and polydispersity index (PdI) for one month, as determined by PCS.

Time (Day)	Plain-LP		LP-MC		LP-MH	
	Mean Size (nm ± s.d.)	PdI ± s.d.	Mean Size (nm ± s.d.)	PdI ± s.d.	Mean Size (nm ± s.d.)	PdI ± s.d.
0	176.9 ± 3.3	0.09 ± 0.02	172.9 ± 2.0	0.10 ± 0.01	209.0 ± 3.0	0.11 ± 0.04
7	174.0 ± 5.4	0.10 ± 0.01	179.2 ± 1.2	0.11 ± 0.01	204.0 ± 3.4	0.10 ± 0.01
15	170.9 ± 3.2	0.12 ± 0.01	177.7 ± 4.3	0.10 ± 0.01	205.9 ± 8.4	0.10 ± 0.03
21	176.5 ± 5.1	0.13 ± 0.01	179.3 ± 5.9	0.12 ± 0.01	207.3 ± 9.4	0.08 ± 0.01
30	171.0 ± 4.8	0.11 ± 0.01	178.2 ± 5.7	0.09 ± 0.01	216.6 ± 7.4	0.18 ± 0.06

**Table 4:** Liposome mean size and polydispersity index (PdI) in FBS, after extrusion and 3 h after dispersion, as determined by PCS.

Dispersion Acronym	FBS after Extrusion		FBS after 3 h	
	Mean Size (nm ± s.d.)	PdI ± s.d.	Mean Size (nm ± s.d.)	PdI ± s.d.
**plain-LP**	180.57 ± 3.69	0.122 ± 0.022	180.77 ± 2.02	0.132 ± 0.005
**LP-MC**	201.47 ± 5.71	0.229 ± 0.016	209.67 ± 2.21	0.206 ± 0.026
**LP-MH**	184.17 ± 4.56	0.135 ± 0.022	182.63 ± 4.89	0.109 ± 0.035

**Table 5:** r1 values (expressed in mM^−1^s^−1^) of the Mn compounds in H_2_O or in FBS either as free or liposome forms as measured at 20 °C and 37 °C.

Compound	H_2_O				FBS			
	r1	R^2^	r1	R^2^	r1	R^2^	r1	R^2^
	20 °C		37 °C		20 °C		37 °C	
MC	7.7	1.000	5.0	1.000	25.8	0.912	22.1	0.914
MH	7.3	0.996	4.6	0.996	25.4	0.969	22.6	0.954
LP-MC	23.5	0.964	16.8	0.989	26.1	0.971	22.3	0.970
LP-MH	20.9	0.963	15.8	0.999	24.1	0.943	19.1	0.930

## Data Availability

Upon reasonable request, data can be obtained from the corresponding author.

## Supplementary Materials

The following supporting information can be downloaded at https://www.mdpi.com/article/10.3390/molecules29143407/s1. Figure S1: HUVEC cell viability evaluated by MTT test. Cells were treated for 24 h with the indicated doses of free-Mn(II)-based contrast agents in aqueous solutions (Sol-MC and Sol-MH). The % of cell viability is expressed with respect to untreated control cells (ctrl). Data are the mean ± SD of three independent experiments with at least three technical replicates each time.
