# Supplementary material for: Manganese-Loaded Liposomes: An In Vitro Study for Possible Diagnostic Application
Source: Molecules. 2024 Jul 20;29(14):3407. doi: 10.3390/molecules29143407 (PMC11280348; doi:10.3390/molecules29143407)
Supplement: Supplementary file 1 [file molecules-29-03407-s001.zip › molecules-3065458-supplementary.pdf]

Supplementary Figure S1

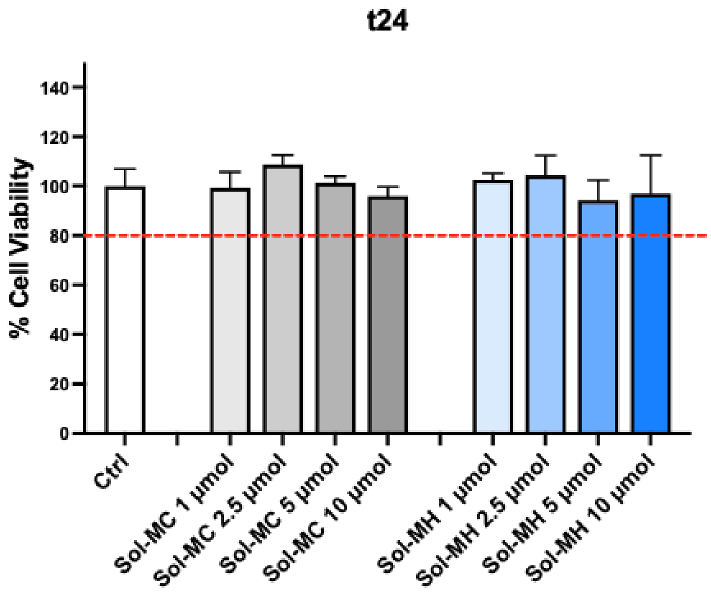

Figure S1. HUVEC cells viability evaluated by MTT test. Cells were treated for 24 h with the indicated doses of free Mn(II)-based contrast agents in aqueous solutions (Sol-MC and Sol-MH). The % of cell viability is expressed with respect to untreated control cells (ctrl). Data are the mean ± SD of three independent experiments with at least three technical replicates each time. (original figure)
